# Data-Driven Optimization of Healthcare Recommender System Retraining Pipelines in MLOps with Wearable IoT Data

**DOI:** 10.3390/s25206369

**Published:** 2025-10-15

**Authors:** Yohan Park, Jonghyeok Mun, Yejung Lee, Jihwan Um, Jongsun Choi, Jaeyoung Choi

**Affiliations:** School of Computer Science and Engineering, Soongsil University, 369 Sangdo-Ro, Dongjak-Gu, Seoul 06978, Republic of Korea; imjin3027@soongsil.ac.kr (Y.P.); jonghyeokmun@soongsil.ac.kr (J.M.); yejung@soongsil.ac.kr (Y.L.); umjh@soongsil.ac.kr (J.U.); choi@ssu.ac.kr (J.C.)

**Keywords:** dynamic data management, data reduction, feature selection, MLOps, model retraining, recommender system

## Abstract

Personalized healthcare recommender systems are increasingly being deployed in edge AI environments through wearable devices. In such environments, cloud servers leverage high-performance GPUs to train base models, which are then optimized for data reduction deployment on edge devices, enabling the delivery of personalized services. However, the base model may experience a gradual decline in accuracy over time, a phenomenon known as model drift. Recommender systems that do not keep up with changes in user preferences risk generating predictions based on outdated behavior, which can negatively impact the user experience. Therefore, it is essential to adopt retraining approaches that incorporate both past training data and new data from wearable devices. To address the drift problem, we propose a dynamic data management strategy, integrated into an automated training pipeline based on machine learning operations (MLOps). This approach enables adaptive model updates in response to continuously evolving IoT data. To preserve base model performance, our strategy leverages data reduction and feature selection algorithms. By dynamically managing data with these techniques, we effectively mitigate data drift and enhance resource efficiency during model retraining. We validated our approach through experiments on personalized fitness recommendations using FitRec wearable data from 1104 users, achieving improved computational efficiency during retraining while preserving model accuracy. Consequently, our dynamic data management method ensures faster training and the sustained performance of data reduction base models essential for edge AI applications. Moreover, this approach presents a compelling solution for continuously refining personalized recommendation services in alignment with evolving user preferences.

## 1. Introduction

Machine learning (ML) has become a foundational technology that underpins advancements across diverse industries and academic disciplines. Among its many applications, recommender systems have received significant attention, with a large body of research aiming to enhance user experience through improved personalization and service efficiency [1]. Recent research has focused on understanding the interactions between users, items, and contextual features to better predict user preferences and enhance recommendation accuracy [2]. These personalized recommender systems are increasingly being applied in high-stakes domains such as healthcare, where reliability is critical. Current research explores their use in personalized health management and broader clinical applications [3]. In particular, the healthcare domain demands not only real-time responsiveness but also stringent protection of personal data. To address these requirements, researchers are increasingly turning to edge environments—such as wearable devices and mobile platforms—where data can be processed locally. This shift away from exclusive reliance on cloud infrastructure enables edge AI to deliver personalized services with enhanced speed, reliability, and security. Within these edge environments, developing effective model update strategies has become essential for maintaining long-term performance and personalization.

Despite these advantages, ensuring the consistent performance of recommender system models deployed on edge devices remains a considerable challenge. One of the most pressing concerns is model drift, a phenomenon in which model accuracy progressively declines over time [4]. This decline primarily results from discrepancies between the data used for initial training and the continuously evolving data collected from wearable devices during real-world operation. Model drift is typically regarded as the result of two key forms of change: data drift and concept drift. Data drift refers to temporal shifts in the statistical distribution of input features, whereas concept drift captures changes in the underlying relationships between inputs and outputs. Without effective retraining mechanisms to address these drifts, recommender systems risk degraded performance, leading to outdated predictions, reduced user trust, and potentially serious errors in healthcare decision support.

For healthcare recommender systems to be effective, both data drift and concept drift must be systematically addressed [5]. As new information accumulates and users’ health conditions and preferences evolve over time, model drift inevitably becomes a recurring challenge. Because healthcare applications directly influence patient outcomes, models must remain highly responsive to drift and adaptable to ongoing changes in health status and behavioral patterns [6].

Addressing these challenges has become the focus of active research on the continuous maintenance of machine learning models. This body of research emphasizes the design of frameworks that automate the development, deployment, monitoring, and maintenance of ML models, thereby ensuring that models sustain optimal performance over time [7,8]. Traditional ML systems, which depend on static datasets and one-time training, are inherently limited in capturing evolving data distributions and shifting user preferences. In contrast, MLOps environments continuously detect changes in data and models, automatically triggering retraining at appropriate intervals to ensure that models flexibly adapt to dynamic healthcare environments [9].

This study aims to develop an efficient strategy for managing ML models in cloud environments. We focus on personalized healthcare recommender systems and propose an MLOps-based strategy designed to mitigate both data drift and concept drift effectively. Our approach, termed Dynamic Data Management (DDM), represents a data-driven optimization method that leverages data versioning to systematically capture shifts in data distributions and their interdependencies. Ultimately, our goal is to preserve model performance while ensuring the reliable delivery of personalized healthcare services.

## 2. Related Work

This section lays the groundwork for our proposed approach by first reviewing the fundamentals of MLOps and recommender systems. We then examine prior research addressing the critical challenges of data drift and retraining efficiency. This review contextualizes our study, which builds upon our earlier work on data reduction and feature selection by integrating these methods into a unified Dynamic Data Management (DDM) strategy within the MLOps framework.

### 2.1. Preliminary

#### 2.1.1. Recommender Systems

Recommender systems, which deliver personalized content by analyzing user behavior, contend with the significant challenge of adapting to dynamic environments. The core issue is model drift, where performance degrades over time due to shifting user preferences and data distributions. To mitigate this, prior research has explored various adaptive strategies. For instance, Sritrakool et al. [10] proposed a system that leverages historical data during preference shifts to infer users updated interests. Sun et al. [11] developed an algorithm that prioritizes the most recently consumed items, operating on the insight that recent activities are stronger indicators of current user interests. Furthermore, other studies have examined approaches that exploit temporal information to track evolving user item relationships, particularly in environments characterized by the continual influx of new users and items [12].

However, while these methods effectively incorporate temporal dynamics, their narrow focus on specific user item interactions often overlooks the broader, systemic challenges of managing the entire retraining pipeline. Consequently, critical issues such as computational inefficiency and data redundancy remain largely unaddressed. Our proposed DDM strategy addresses this gap by offering a holistic framework that not only captures temporal variations but also systematically optimizes for data scale and quality, creating a more robust and efficient solution for continuous model maintenance.

#### 2.1.2. Edge MLOps

**Figure 1:**
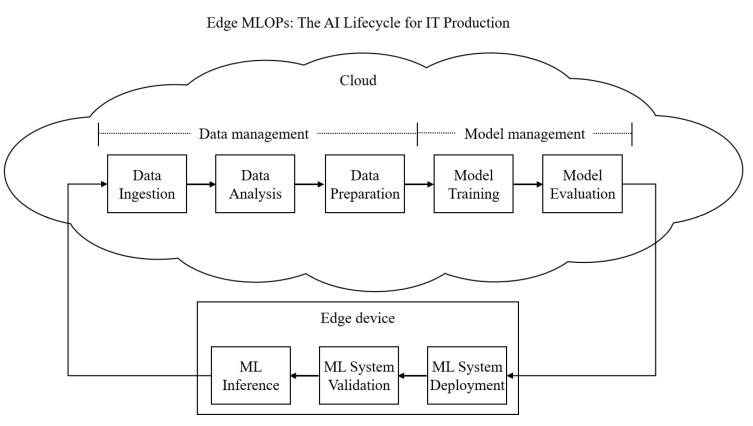
Overall lifecycle of Edge MLOps.

Edge MLOps automates the machine learning pipeline—from data ingestion to deployment—while clearly delineating the distinct functional roles of cloud servers and edge devices. As illustrated in Figure 1, a representative workflow involves training models in the cloud and subsequently deploying them to edge devices where inference services are delivered to users [13]. This paradigm is designed to support continuous model improvement. For instance, Bayram et al. [14] proposed a strategy that leverages a feedback loop to continuously monitor model performance and trigger updates whenever degradation is detected.

Operationalizing these updates effectively hinges on two critical decisions, as highlighted in prior research [15]: determining when to retrain and selecting how the model should be updated. The former is often governed by predefined thresholds based on performance degradation or data distribution shifts, while the latter involves choosing between strategies like continuous learning and periodic retraining to mitigate model drift. Our study contributes to this line of research by focusing on the “how”, proposing an efficient data management strategy to optimize the data pipelines that underpin effective model updates.

### 2.2. Model Retraining and Continuous Learning

Machine learning models are typically updated using one of two primary approaches: continuous learning or periodic retraining. While continuous learning is suited for real-time applications, periodic retraining is often a more cost-efficient strategy in MLOps environments. Mahadevan et al. [16] underscored this point, arguing that overly frequent retraining imposes unnecessary computational costs and advocating for cost-aware strategies. However, this approach introduces its own challenges, namely the accumulation of redundant data and catastrophic forgetting, where new information overwrites previously learned patterns [17].

Prior work has sought to remedy these issues through various methods. Data-driven approaches include strategies that dynamically regulate the volume of past data to reduce inefficiencies [18] or, as Kirkpatrick et al. [19] introduced, methods that adjust the weight of past data to prioritize new information while retaining earlier patterns. In parallel, model-driven techniques have been proposed, such as selectively updating specific neural network layers to minimize the erosion of prior knowledge [20]. Although effective in isolation, these strategies typically fail to synthesize a solution that addresses both data volume and data quality concurrently. This study bridges this critical gap by proposing a DDM framework that unifies data reduction and feature selection. By systematically filtering redundant records while retaining high-importance features, our approach simultaneously enhances computational efficiency and mitigates catastrophic forgetting, offering a more robust solution for maintaining model performance in dynamic healthcare settings.

### 2.3. Data Management in ML

**Figure 2:**
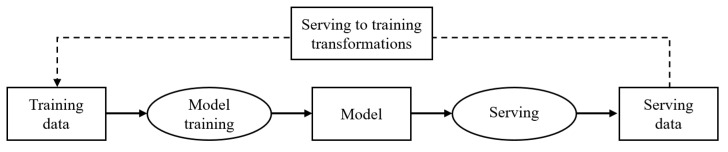
Simplified production machine learning pipeline (rectangles represent data artifacts, while ellipses denote processes).

As machine learning becomes increasingly integrated into real-world services, robust data management across the entire data lifecycle has become pivotal. The management of preprocessing tasks like data exploration, cleansing, and labeling directly influences model performance and stability [21,22]. MLOps frameworks address this by leveraging automated pipelines to manage evolving data, as illustrated in Figure 2. These pipelines are designed to handle incoming data efficiently by reducing inter-dataset dependencies and discarding redundant information [23], which has been empirically shown to mitigate data drift and stabilize model performance [24].

Prior work in dynamic data management has explored various specific methods. For instance, Baumann et al. [25] demonstrated a technique that dynamically adjusts the balance between historical and new data, thereby preserving historical patterns while adapting to new information. Other studies have emphasized the importance of data quality over quantity, particularly for complex healthcare datasets where time-series and imbalanced characteristics demand a focus on the completeness of data rather than sheer volume [26]. However, these efforts have treated data volume and quality as disparate problems, often addressing one in isolation without a unified framework that optimizes both.

This study bridges this gap by proposing a novel strategy that unifies data reduction and feature selection into a single, cohesive framework. Our approach produces optimized data versions by simultaneously reducing redundant historical data to manage scale and selecting salient features to enhance quality and interpretability. This dual-action method not only sustains model performance in dynamic environments but also significantly reduces the computational overhead of retraining. Consequently, our framework presents a more comprehensive and practical solution for continuous model maintenance, a critical requirement for efficient Edge AI applications.

### 2.4. Data Reduction

The continuous accumulation of data in machine learning systems presents the dual challenges of increased overfitting risk and amplified computational costs. Prior work has established that strategically removing redundant data can yield clearer outcomes [27] without significantly compromising model performance [28]. Consequently, various data reduction methods have been explored. For example, Zhang et al. [29] employed a matrix factorization-based strategy to minimize computational costs, whereas Niu et al. [30] and Ahmadian et al. [31] proposed temporal data reduction approaches to improve accuracy. Our data reduction module evolved from techniques validated in our previous research [32], which confirmed that combining matrix factorization with similarity-based methods can mitigate overfitting while substantially lowering the computational load.

However, a critical limitation of these conventional approaches is their singular focus on maximizing data volume reduction, often neglecting the potential loss of vital information. This narrow focus imposes a detrimental trade-off, where gains in computational efficiency are achieved at the expense of model accuracy, as the reduced dataset may no longer represent the underlying data distribution. Our proposed framework directly counteracts this limitation. By integrating a feature selection mechanism alongside data reduction, our strategy not only manages data volume but also actively preserves data quality. This synergistic approach forges a more robust and effective solution for maintaining stable model performance in dynamic MLOps environments.

### 2.5. Feature Selection

Feature selection is a critical discipline within machine learning and data mining that enhances model performance and computational efficiency by identifying the most salient variables in a dataset [33]. By eliminating irrelevant features, this process is particularly effective for improving training outcomes in high-dimensional data and mitigating overfitting [34]. Conventional techniques are typically categorized as Filter, Wrapper, or Embedded methods, which have demonstrated superiority over the indiscriminate use of all available features [35]. However, these approaches often suffer from a fundamental limitation: they operate independently of the final recommendation model. This creates a suboptimal alignment between the selected features and the model’s predictive objectives, a gap that recent AutoML-based methods have sought to address [36].

Our framework circumvents this limitation by extending a model-aware controller validated in our prior work [37]. That study proposed a controller that integrates the recommender’s outputs with data analysis results to dynamically derive user-specific feature weights, thereby improving both predictive performance and interpretability. The central contribution of this study is to unify this adaptive feature selection module with our data reduction module [32], making them the cornerstone of our DDM strategy. These two components work synergistically; the data reduction module manages computational load and overfitting [32], while the feature selection module ensures that only the most informative, user-specific features are retained [37]. This integrated approach allows our system to proactively combat data and concept drift while efficiently maintaining performance and interpretability during retraining.

## 3. Data-Driven Optimization with DDM

We propose a Data-Driven Optimization approach, termed DDM, for healthcare recommender systems in Edge MLOps environments. This approach streamlines model retraining while adapting to both data drift and concept drift. Built on validated techniques of data reduction and feature selection, DDM generates dynamic data versions that are seamlessly integrated into the MLOps pipeline.

**Figure 3:**
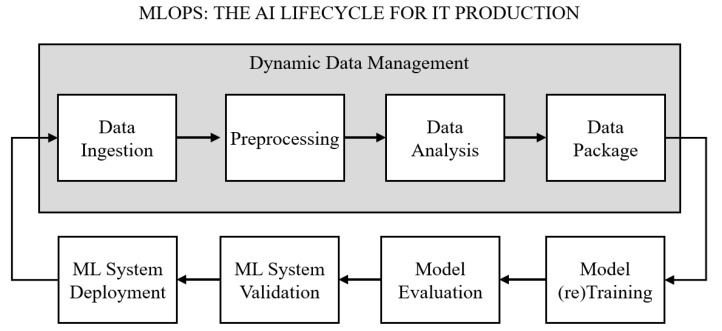
Overall architecture of the proposed Data-Driven Optimization framework based on DDM.

This integration not only preserves model performance but also improves computational efficiency in practice. As illustrated in Figure 3, the framework operates in iterative cycles: data are ingested, processed, and analyzed to produce new versions, which subsequently drive model training, evaluation, and deployment. Each cycle generates refined data versions that ensure retraining remains responsive to evolving data patterns and that system performance is consistently sustained.

### 3.1. Overview of DDM

**Figure 4:**
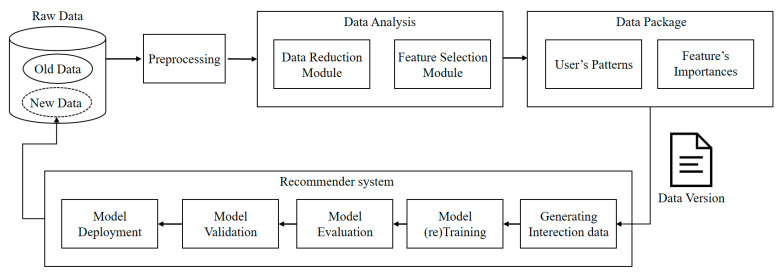
Workflow of dynamic data versioning for recommender systems using feature selection and data reduction.

DDM is designed to sustain robust model performance in MLOps environments by ensuring stability under continuously evolving datasets. As illustrated in Figure 4, the architecture adopts a data-driven approach that integrates feature selection and data reduction modules to construct optimized data versions. These versions balance the preservation of historical user behavior patterns with adaptation to data drift. In doing so, the framework seamlessly incorporates shifting data while sustaining a consistent user experience. The generation of dynamic data versions is organized into four stages: raw data ingestion, preprocessing, data analysis, and data packaging.

The raw dataset consists of both previously trained data and newly collected serving data, the latter generated directly from service interactions. During preprocessing, essential refinement steps—such as removing missing values and duplicates—are performed to ensure data integrity and reliability. The analysis phase incorporates two complementary modules:

- Feature selection module: identifies and retains the most critical user-relevant features, preserving informative attributes essential for effective model training.
- Data reduction module: implemented using matrix factorization, eliminates redundant portions of the training data, thereby compressing the dataset and improving computational efficiency.

The data packaging stage consolidates the outcomes of feature selection and data reduction to produce a dynamic data version. In this stage, redundant historical records are removed while essential behavioral patterns are preserved, and user-specific salient features are incorporated to improve data quality. The resulting packaged dataset is stored as a new version and supplied to the MLOps pipeline, where it supports efficient retraining and evaluation.

By integrating these modules, the framework generates well-balanced data versions that retain historical behavioral patterns while incorporating newly emerging data. This enables proactive adaptation to both data drift and concept drift, preventing performance degradation and ensuring model stability.

### 3.2. Raw Data

In constructing dynamic data versions, raw data are categorized into two types:

- Old Data: datasets previously used for training recommender models, which preserve historical patterns of user behavior.
- New Data: data collected during service operation, which capture the most recent user behavior.

Healthcare wearable data—such as heart rate, exercise intensity, and recovery rate—are highly variable over time. Therefore, integrating Old and New Data is essential for mitigating model drift.

### 3.3. Preprocessing

During preprocessing, dataset reliability is ensured by removing missing values, eliminating duplicates, and aligning user and item identifiers. Beyond these standard procedures, the system extracts domain-specific features from wearable sensor logs. For instance, exercise duration is derived from start and end timestamps, and total distance is calculated using speed and GPS coordinates. Physiological metrics such as peak heart rate and recovery speed, which reflect post-exercise stabilization, are also incorporated.

### 3.4. Data Analysis

The procedure of DDM is summarized in Algorithm 1. Data are analyzed through the data reduction and feature selection modules, which subsequently generate data versions serving as inputs for machine learning training.
**Algorithm 1:** Dynamic Data Management (DDM) Workflow1**Input**: New_data (csv), Old_data (csv)2**Output**: Data_version_N (csv)

3NewData, OldData ← read_csv(New_data), read_csv(Old_data)4**function** dynamic_data_management(OldData, NewData):5  NewData_preprocessed ← preprocess(NewData)6  UserPatternData, FeatureImportances ← analyze_data(OldData, NewData_preprocessed)7  DataVersion_N ← package_data(UserPatternData, NewData_preprocessed, FeatureImportances)8  **return** DataVersion_N9**end function**

10**function** analyze_data(OldData, NewData_preprocessed):11  UserPatternData ← data_reduction_module(OldData)12  FeatureImportances ← feature_selection_module(UserPatternData, NewData_preprocessed)13  **return** UserPatternData, FeatureImportances14**end function**

15**function** package_data(UserPatternData, NewData_preprocessed, FeatureImportances):16  DataVersion_N ← combine(UserPatternData, NewData_preprocessed)17  DataVersion_N ← apply_weights_and_filter (DataVersion_N, FeatureImportances)18  **return** DataVersion_N19**end function**

20**function** data_reduction_module(data)21  SummarizedData ← summarize_time_series_features(data)22  perSportData ← filter_by_sports_type(SummarizedData)23  ReducedData ← apply_pca(perSportData)24  UserPatternData ← analyze_similarity(ReducedData)25  **return** UserPatternData26**end function**

27**function** feature_selection_module(UserPatternData, NewData_preprocessed)28  EmbeddedFeatures ← combine_features(attributeFeatures, contextualFeatures, sequentialFeatures, healthMetricFeatures)29  FeatureWeights ← calculate_feature_weights(EmbeddedFeatures)30  FeatureImportance ← select_important_features(FeatureWeights)31  **return** FeatureImportance32**end function**

As outlined in Algorithm 1, the DDM workflow is engineered to optimize the retraining pipeline by systematically managing data volume and quality. The process commences with the ingestion of historical (OldData) and newly collected (NewData) datasets. The main dynamic_data_management function orchestrates the entire pipeline, beginning with the preprocessing of NewData. It then invokes the analyze_data function, which executes a dual analysis: the data_reduction_module first distills OldData into UserPatternData, a compressed representation of essential user behaviors. Subsequently, the feature_selection_module evaluates features from both this historical summary and the new data to compute their predictive importance (FeatureImportances). In the final stage, the package_data function synthesizes the new data version by merging UserPatternData with the preprocessed new data and then refining the combined dataset using the calculated FeatureImportances. This systematic process ensures that the final output is both resource-efficient and information-rich, enabling the system to proactively address data drift while sustaining model performance.

#### 3.4.1. Data Reduction Module

The data reduction module extracts latent temporal features and computes cosine similarity across users to construct a similarity matrix. When user pairs exceed a predefined threshold, redundant sessions with fewer items are removed, thereby lowering dataset complexity without discarding essential information.

**Figure 5:**
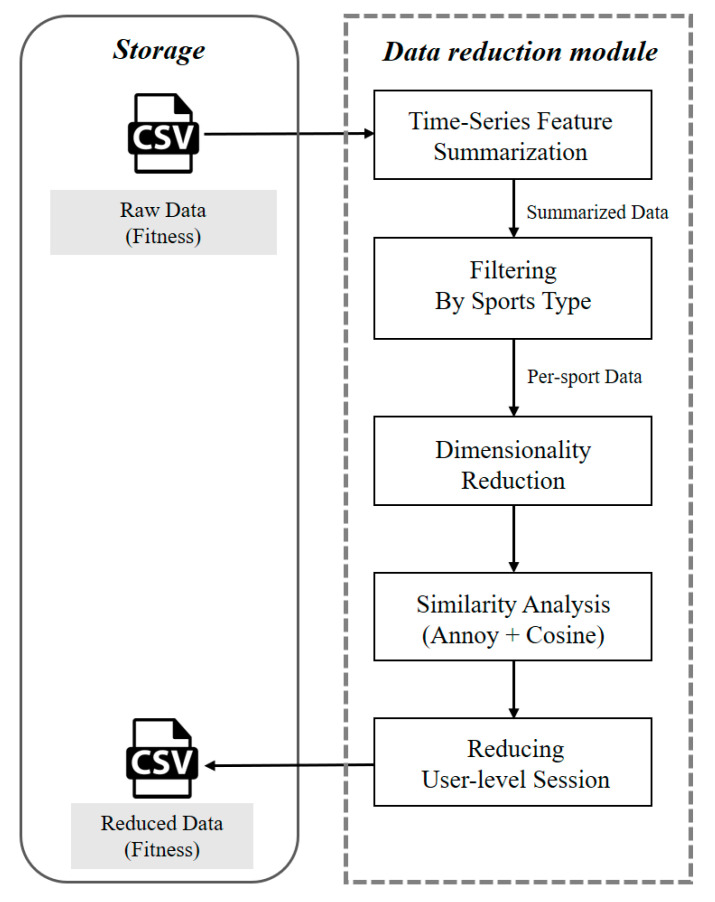
The Data Reduction Module Workflow. The five-stage pipeline transforms raw time-series exercise data into a condensed, optimized dataset. The process involves (1) feature summarization, (2) filtering by sports type, (3) dimensionality reduction, (4) similarity analysis, and (5) user-level session reduction. This approach is designed to enhance computational efficiency while preserving essential user behavior patterns.

As illustrated in Figure 5, the module operates in five sequential stages, with time-series exercise log data serving as both input and output:

- **Time-Series Feature Summarization**: Each session is condensed into a fixed-length vector using statistical measures such as mean, standard deviation, minimum, and maximum. This reduces the computational burden of analyzing raw sequences.
- **Filtering by Sports Type**: The distribution of activities is analyzed, and sports categories with sufficient data volume are selectively retained. This reduces heterogeneity across exercise patterns (e.g., varying heart rate profiles) and enhances analytical reliability.
- **Dimensionality Reduction**: Principal Component Analysis (PCA) is applied to the selected activity-specific data to reduce the feature space while preserving key information and improving the efficiency of similarity assessment.
- **Similarity Analysis**: Reduced vectors are indexed using Annoy, and cosine similarity is applied to enable efficient large-scale comparisons of session-level exercise patterns.
- **Reducing User-Level Sessions**: For each user’s dataset, highly similar sessions are consolidated by retaining a single representative instance, thereby reducing redundancy while preserving the integrity of user-specific distributions.

Through this process, the dataset size is substantially reduced while essential behavioral patterns are retained. As a result, the generated training data strike a balance between computational efficiency and informational richness.

#### 3.4.2. Feature Selection Module

**Figure 6:**
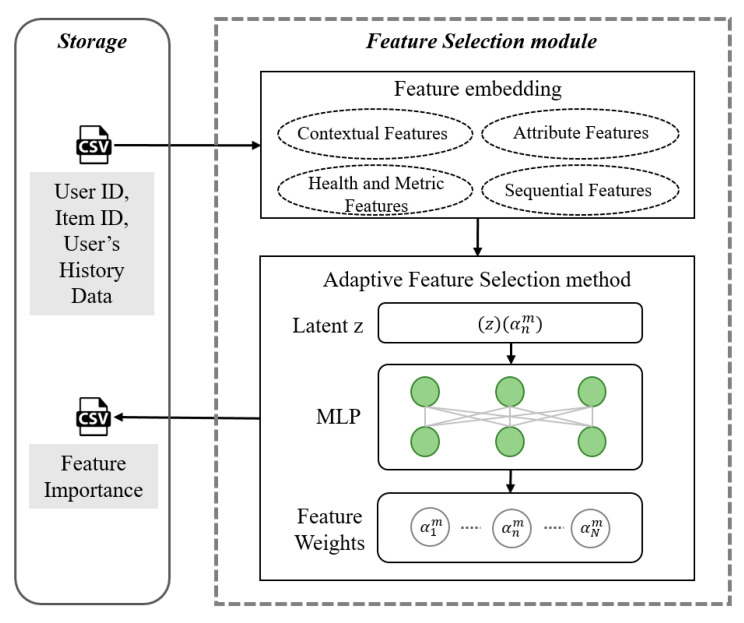
The feature selection module workflow. The module operates in two stages: (1) Feature Embedding, where user and item data are transformed into categorized features, and (2) Adaptive Feature Selection, where a model-aware MLP controller, whose hidden layer neurons are depicted as green circles, dynamically computes feature weights ($a_{n}^{m}$) based on a latent vector z. This process identifies the most critical features to enhance both predictive performance and model interpretability.

The feature selection module employs a model-aware controller to dynamically assign importance weights to input features. As shown in Figure 6, the module first categorizes embedded user, item, and historical log data into four distinct groups:

- **Attribute Features**: user-related attributes.
- **Contextual Features**: exercise-related context such as intensity, type, and recovery status.
- **Health and Metric Features**: physiological measures including heart rate and blood pressure.
- **Sequential Features**: temporally ordered activity patterns.

At its core, a multi-layer perceptron (MLP) controller governs the weighting process, assigning a weight $a_{n}^{m}$ to each feature based on a latent vector *z* to prioritize the most impactful signals for recommendation. The controller’s design is informed by state-of-the-art AutoML frameworks [38] and consists of a two-layer MLP with ReLU activation, a bi-level optimization strategy that uses validation loss as a supervision signal, and dropout regularization (rate = 0.2). The effectiveness of this approach has been previously validated in our prior work, where it was proven competitive against strong baselines like AdaFS and AutoField [38].

#### 3.4.3. Data Package

The DDM workflow culminates in the Data Package stage, where the outputs from the preceding modules are synthesized into a final, optimized data version. This process begins by merging the compressed historical data (UserPatternData) with the preprocessed new data (NewData_preprocessed). This aggregated dataset is then refined by applying the FeatureImportances to selectively filter or weight the most critical features. The result is a strategically balanced dataset that reduces computational load while preserving high informational value, ensuring the system can effectively adapt to data drift and maintain stable performance during retraining.

### 3.5. Implementation of the MLOps Environment

All experiments were conducted on a server equipped with an Intel Core i9-10920X CPU (Intel, Santa Clara, CA, USA), 64 GB of RAM, and two NVIDIA GeForce RTX 4080 (16 GB) GPUs (NVIDIA, Santa Clara, CA, USA).

Our MLOps pipeline, orchestrated by Apache Airflow, integrates with a Kubernetes cluster. This integration is enabled by the apache-airflow-providers-cncf-kubernetes package, which facilitates the use of the KubernetesExecutor. This executor dynamically launches each DAG (Directed Acyclic Graph) task as an independent Kubernetes pod, an architecture that prevents resource contention and supports dynamic scaling.

We implemented each pipeline stage—including data reduction and model retraining—as a distinct task using the KubernetesPodOperator. This operator allows for the creation of a dedicated pod for each task, enabling us to define task-specific Docker images and environmental configurations. This modular approach enhances the flexibility and reliability of the entire MLOps pipeline.

The MLOps pipeline was orchestrated by Apache Airflow atop a Kubernetes cluster. This architecture leverages the apache-airflow-providers-cncf-kubernetes package, which enables the Kubernetes Executor to dynamically instantiate each DAG task as an independent Kubernetes pod. This design prevents resource contention and supports dynamic scaling. Each stage of our workflow, from data reduction to model retraining, was implemented as a distinct task using the KubernetesPodOperator. This approach encapsulates each task within a dedicated pod with its own Docker image and environment, enhancing the pipeline’s modularity and reliability. Table 1 provides a comprehensive list of the software packages and versions used in our experiments.

The proposed DDM system synergistically integrates data reduction and feature selection to achieve a dual objective: it eliminates data redundancy to enhance computational efficiency while preserving high-importance features to maintain model accuracy. This design is particularly crucial in domains like healthcare, where clinically vital but highly variable signals—such as heart rate and recovery speed—must be retained to ensure reliable performance. By uniting these principles within an automated MLOps pipeline, our framework yields a system that is both resource-efficient and robust, capable of delivering continuous, stable performance in real-world service environments.

## 4. Experiments and Results

This study evaluates the proposed DDM system with a focus on its effectiveness in reducing computational overhead and sustaining stable recommender model performance during retraining within an MLOps environment. The experimental framework follows a structured process consisting of data preparation, environment configuration, algorithm implementation, comparative evaluation, performance metric specification, and result analysis. For this purpose, we employ the FitRec dataset, which contains wearable exercise records from 1104 users across multiple activities, including running, cycling, and walking. Using this dataset, we examine whether the DDM strategy improves retraining efficiency compared to conventional static data management approaches.

### 4.1. Experimental Environment

**Figure 7:**
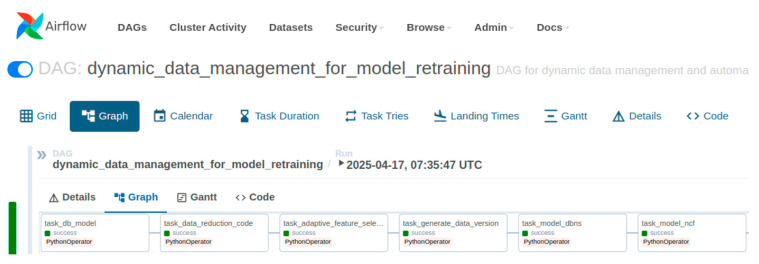
The Automated Retraining Pipeline as an Apache Airflow DAG. This diagram visualizes the retraining workflow, a core, operational component of the broader MLOps lifecycle illustrated in Figure 1. Each node in the Directed Acyclic Graph (DAG) encapsulates a specific, containerized task—such as data reduction or model evaluation—that is executed sequentially. This automated structure is instrumental in delivering stable and timely model updates in a real-world service environment.

The retraining pipeline, illustrated in Figure 7, is implemented within the MLOps environment and orchestrated by Apache Airflow. It automates and manages the entire workflow, from data collection to model updates. To ensure environmental consistency, Docker is used to containerize each stage of the pipeline, with data preprocessing, dynamic data generation, model training, and evaluation executed seamlessly within individual containers. Data management is supported by MariaDB, where dynamic data versions—produced through data reduction and feature selection algorithms—are stored and subsequently used as inputs for training and evaluation. For model implementation, we employ an LSTM-based Deep Belief Network (DBN) model alongside a deep learning based Neural Collaborative Filtering (NCF) model, both retrained periodically under the orchestration of Airflow.

### 4.2. Experimental Data

The experiments are conducted using the FitRec dataset, which contains 253,020 exercise sessions from 1104 users. This dataset encompasses 49 different exercise types—including running, cycling, and walking—and includes wearable measurements such as speed, altitude, and GPS coordinates [39]. For analysis, the data are divided into two categories: Old Data, used in prior model training to preserve historical patterns, and New Data, collected during service operation to capture recent behavior.

To simulate a continuous retraining scenario, the dataset is divided chronologically into four stages:

- **Stage 1 (Initial Training)**: 55% of the earliest sessions, used to train the initial model.
- **Stage 2 (First Retraining)**: the Stage 1 model is retrained on 55% old data + 15% new data, where old data preserves historical patterns and new data reflects recent user behavior.
- **Stage 3 (Second Retraining)**: the model is updated with 70% old data + 15% new data.
- **Stage 4 (Third Retraining)**: the model is updated with 85% old data + 15% new data.

Recent studies suggest that immediate retraining upon the arrival of new data is not always optimal. Zanotti et al. [40] demonstrated that periodic retraining using accumulated data, rather than continuous updates, sustains predictive performance while substantially reducing computational costs. Similarly, Bertsimas et al. [41] showed that aggregating data for scheduled retraining, instead of incorporating every instance in real time, mitigates model instability and enhances interpretability.

Building on these findings, this study adopts a strategy of retraining once a sufficient volume of new data has accumulated. This is considered a balanced approach in terms of accuracy, efficiency, and reliability. Moreover, this setting reflects the practical characteristics of healthcare environments, where patient data are periodically collected and integrated.

### 4.3. Evaluation Metrics

To comprehensively evaluate the effectiveness of the proposed DDM strategy, we consider three dimensions: prediction accuracy, recommendation accuracy, and resource efficiency. This framework reflects the fact that retraining in healthcare environments must deliver not only accurate predictions but also efficient and timely operations.

#### 4.3.1. Metrics for Time-Series Prediction

Time-series prediction performance is assessed using MAE (Mean Absolute Error) and RMSE (Root Mean Squared Error). MAE quantifies the average absolute deviation between observed and predicted values, with lower scores indicating superior performance. RMSE, by amplifying the effect of larger errors through squared deviations, is particularly well suited for capturing instability caused by model drift.

(1) $$MAE=\frac{1}{n}\sum_{i=1}^{n}\left|y_{i}-{\hat{y}}_{i}\right|,$$

(2) $$RMSE=\sqrt{\frac{1}{n}\sum_{i=1}^{n}{\left(y_{i}-{\hat{y}}_{i}\right)}^{2}}.$$

#### 4.3.2. Metrics for Recommendation Accuracy

Recommendation accuracy is evaluated using Recall@K, Precision@K, F1 score@K, and Normalized Discounted Cumulative Gain (NDCG@K). Recall@K measures the proportion of relevant items retrieved within the top-K recommendations. Precision@K quantifies the accuracy of the recommendations by measuring the fraction of items within the top-K list that are actually relevant to the user. The F1 score@K is the harmonic mean of Precision and Recall, providing a single, balanced metric that reflects the overall effectiveness by considering both the accuracy and completeness of the recommendation set. Finally, NDCG@K incorporates ranking positions by assigning higher scores when relevant items appear closer to the top of the list. In this study, the cutoff value K is consistently fixed at 10 (i.e., K = 10) for all ranking metrics.

(3) $$Recall=\frac{TP}{TP+FN},Precision=\frac{TP}{TP+FP},\;F1\;score=2\times \frac{Precision\;\times \;Recall}{Precision\;+Recall}$$

(4) $$DCG=\sum_{i=1}^{n}\frac{2^{{rel}_{i}}-1}{{log}_{2}(i+1)},$$

(5) $$NDCG=\frac{DCG}{IDCG}.$$

#### 4.3.3. Metrics for Resource Efficiency

Resource efficiency is measured in terms of retraining time under consistent conditions. In MLOps environments, both retraining frequency and processing time directly affect the speed of service delivery. These factors therefore serve as critical indicators for assessing the system’s operational efficiency.

### 4.4. Experimental Results

In this section, we present the experimental findings of the proposed DDM approach in comparison with baseline strategies, including full retraining and simple data merging. The comparison focuses on three dimensions: time-series prediction, recommendation accuracy, and resource efficiency. We also track performance trends across Stages 1 to 4 and interpret the implications of the observed changes in each metric.

Our presentation of the results is structured to first validate the effectiveness of the core DDM components, followed by an end-to-end evaluation of the entire framework. We begin by presenting the outcomes of the data reduction (Section 4.4.1) and feature selection (Section 4.4.2) modules. We then assess the system’s performance on its primary tasks: time-series prediction (Section 4.4.3) and recommendation accuracy (Section 4.4.4). Finally, we analyze the framework’s resource efficiency (Section 4.4.5) and present a detailed ablation study (Section 4.4.6) to verify the contribution of each individual module.

#### 4.4.1. Results of Data Reduction

Across Stages 1 to 4, dataset versions were managed dynamically, resulting in a progressive reduction of Old Data, as summarized in Table 2. The reduction process employed PCA for dimensionality compression and cosine similarity to identify and eliminate redundant records. With the similarity threshold set at 0.99, the recommender model maintained stable performance. Importantly, the threshold can be tuned to balance two objectives: preventing the loss of essential information during reduction and facilitating the smooth integration of new data. The resulting decrease in dataset size not only accelerates retraining by lowering the volume of data to be processed but also minimizes performance loss, since only highly similar sessions are removed.

#### 4.4.2. Results of Feature Selection

The feature selection module refines each data version by extracting the features most critical to model performance and using them as inputs to the recommender system. Within wearable data, heart rate consistently emerges as a key signal—reflecting physiological status, indicating potential health risks, and guiding both exercise type and intensity. Building on this insight, our study centers exercise recommendations around heart rate, leveraging wearable data to improve prediction accuracy. The adaptive algorithm assigns greater weight to features with the strongest predictive impact, revealing that while feature importance shifts slightly across data versions, average heart rate, exercise intensity, and recovery speed consistently stand out as dominant indicators.

#### 4.4.3. Results of Time-Series Prediction

**Figure 8:**
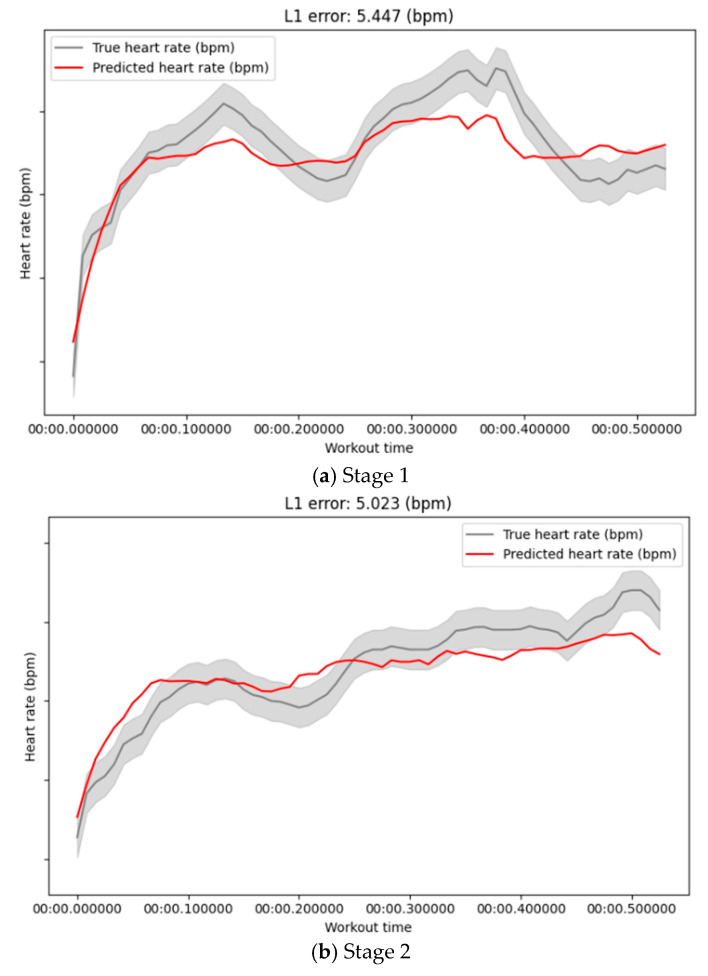

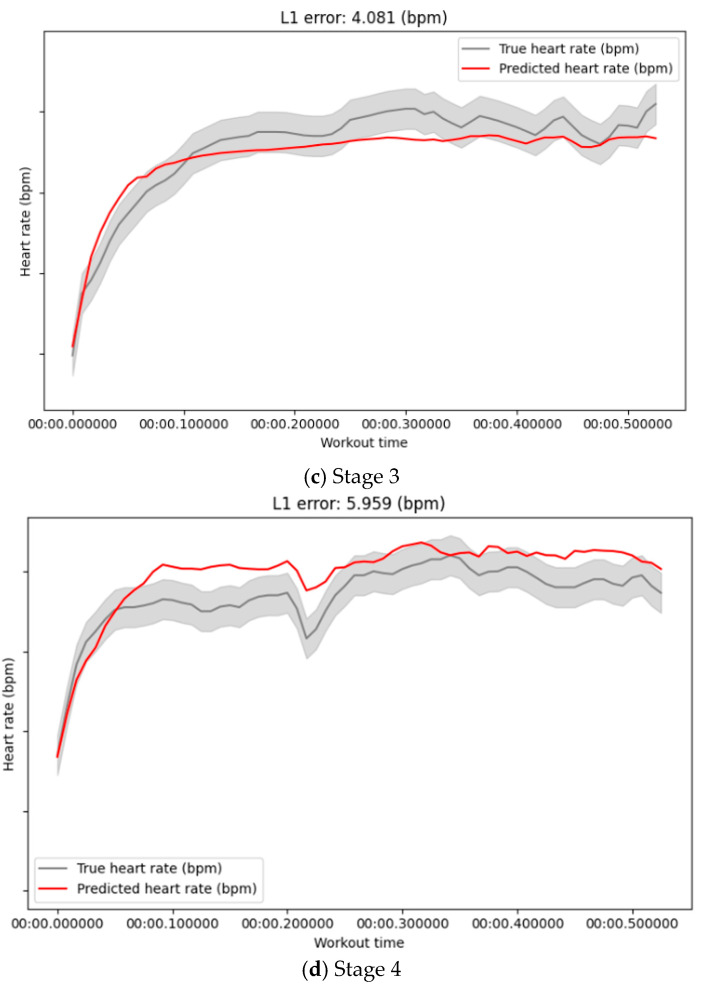
Heart Rate Prediction Across Retraining Stages. This figure compares the true heart rate (red line) with the model’s predicted heart rate (gray line) for each of the four retraining stages (**a**–**d**). The L1 error (MAE) is displayed on each subplot to quantify the predictive accuracy at that stage.

**Figure 9:**
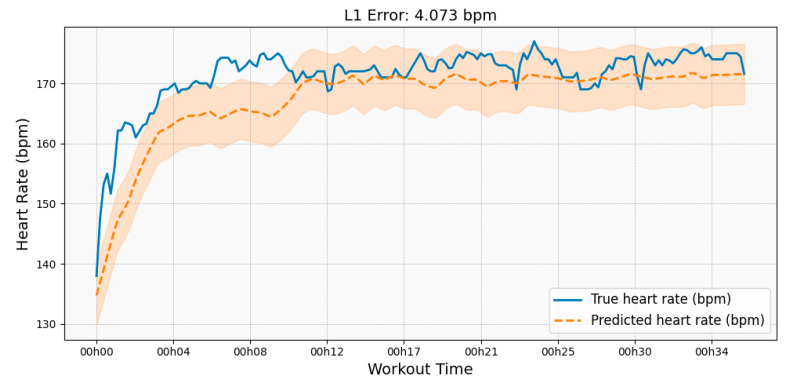
Baseline Heart Rate Prediction on the Full Dataset. This plot illustrates the performance of a model trained on the entire dataset, without the proposed DDM strategy. It serves as the baseline for evaluating the predictive accuracy of the stage-wise DDM approach presented in Figure 9.

The time-series prediction model (DBN) was evaluated using MAE (Mean Absolute Error) and RMSE (Root Mean Squared Error). MAE measures the average prediction error, while RMSE, being more sensitive to large deviations, is particularly useful for assessing stability. As shown in Figure 8 and Figure 9, the results of full training and stage-wise retraining remain broadly consistent, indicating that the DDM strategy preserves prediction performance while reducing computational costs.

Table 3 details the stage-by-stage performance of the time-series prediction model. The initial training at Stage 1 established a baseline MAE of 5.2. Following the first retraining cycle at Stage 2, the MAE temporarily rose to 6.7, reflecting the model’s initial adaptation to new data patterns. However, performance significantly improved by Stage 3, with the MAE decreasing to 4.5 and RMSE to 10.1. The model achieved its most stable and accurate outcomes at Stage 4, delivering a final MAE of 3.9 and an RMSE of 8.7, both of which surpassed the initial baseline performance.

The transient increase in MAE at Stage 2 is characteristic of an initial adaptation phase, where the model adjusts to discrepancies between historical and new data. Subsequently, the model’s performance not only stabilized but significantly improved, with both MAE and RMSE at Stage 4 falling below their initial baselines. This trend validates that our DDM strategy, which combines data reduction and feature selection, effectively enhances predictive accuracy over time. The greater variability observed in RMSE compared to MAE further underscores the metric’s sensitivity to outliers and abrupt shifts in heart rate. Ultimately, these results confirm our approach’s adeptness at navigating volatile real-world data, preserving overall stability while minimizing performance loss during sharp fluctuations

#### 4.4.4. Results of Recommender Model

Our recommendation model, based on Neural Collaborative Filtering (NCF), leverages three key inputs to generate personalized suggestions: user IDs, exercise IDs, and the heart rate values predicted by the DBN model. The predicted heart rate serves as a rich contextual feature to help forecast future exercises a user is likely to perform. During training, we employed a random sampling strategy for negative instances to enhance data augmentation, and all evaluations were conducted on top-10 recommendations (K = 10).

To ensure a robust evaluation and prevent data leakage, we adhered to a strict chronological data splitting protocol. The dataset was sorted by timestamp, with the oldest 80% of interactions used for training, the next 10% for validation, and the most recent 10% for testing. For model training, each positive user-exercise interaction was paired with four negative instances, which were uniformly sampled from the entire item catalog. The ground-truth for evaluation was defined as the set of exercise IDs a user actually consumed in the test set. Finally, we clarify that the heart rate predictions from the DBN model function exclusively as contextual input features for the NCF model; its predictive task is to recommend future exercises, not to predict heart rate values.

The results in Table 4 show a consistent improvement in all recommendation metrics as the retraining stages progress. While this improvement is partly attributable to the natural increase in cumulative training data, the more critical finding lies in the comparison between Stage 4 and the “Full training” baseline.

The performance at Stage 4—achieved using an intelligently reduced dataset—closely mirrors that of the model trained on the entire dataset. For instance, the NDCG score at Stage 4 (0.1480) is nearly identical to the baseline (0.1572). This demonstrates that our DDM strategy successfully achieves its primary goal: it goes beyond merely accumulating data and effectively harmonizes the core patterns of existing users with the latest trends from new data. This result, combined with the significant efficiency gains shown in Table 5, validates that our framework maintains near-optimal recommendation quality while substantially reducing the computational cost of retraining.

#### 4.4.5. Resource Efficiency

Resource efficiency was evaluated by comparing retraining times across methods, as summarized in Table 5. The comparison involves three approaches:

- **Retraining from scratch**: all old and new data are used at every retraining step.
- **Simple data merging**: old and new data are blended in predetermined ratios for retraining.
- **Proposed DDM**: old data are reduced, and adaptively selected key features are retained to generate dynamic data versions for retraining.

The Simple data merging method serves as a crucial benchmark. It is designed to demonstrate that our DDM’s performance gains stem from qualitative data optimization, not just volume reduction. For this baseline, we retrain the model using a dataset where the amount of old data is identical to that of our DDM’s reduced dataset. However, this data is selected via uniform random sampling from the original old data, in contrast to our DDM’s intelligent, pattern-based selection. This ensures a fair comparison of the data selection methodologies.

At Stage 4, the proposed approach reduced retraining time by approximately 32%. By decreasing both dataset size and feature dimensionality, the method directly lowered computational demand, resulting in faster retraining and more efficient resource utilization. Over extended MLOps deployments, these improvements can translate into reduced operational costs and enhanced scalability.

#### 4.4.6. Ablation Study

To justify our hyperparameter selections for the data reduction module, we conducted a comprehensive sensitivity analysis.

This version restructures the paragraph to introduce the analysis, define the baseline, and then discuss the results in a single, logical flow.

We conducted a sensitivity analysis to determine the optimal hyperparameters for our data reduction module. First, we evaluated the impact of the cosine similarity threshold, with the results presented in Table 6. In this table, the “Original” row serves as the baseline for the analysis, representing the performance on the full, unfiltered Stage 4 dataset where no data reduction is applied (Reduction Rate = 0%). This scenario is equivalent to the “Retraining from scratch” baseline in Table 5, which has a retraining time of 31 min.

The analysis demonstrates a clear trade-off between recommendation accuracy (NDCG) and computational efficiency (Reduction Rate, Retraining Time). While a threshold of 99.5% achieves the highest NDCG, we selected 99% as our final value. This choice provides a superior balance, securing a 14% data reduction and a significant decrease in retraining time with only a negligible impact on performance.

Second, for dimensionality reduction, we experimented with the number of PCA components (n = 3, 4, 5, 6), which yielded cumulative explained variance values of 85%, 95%, 98%, and 99%, respectively. We chose n = 5, as it preserves 98% of the variance, ensuring robust feature representation while avoiding the minimal gains and additional computational cost of using n = 6.

Finally, we optimized the Annoy index parameters (n_trees, search_k). A configuration of (10, 100) achieved 99% recall compared to a brute-force search baseline. While increasing the parameters to (100, 1000) improved recall to 99.9%, it also quadrupled the computational cost. We therefore selected (10, 100) as the most efficient configuration.

To isolate and quantify the contribution of each core component, we conducted an ablation study with the results summarized in Table 7. The findings revealed that both the Data Reduction and Feature Selection modules are integral to the framework’s success. Removing the Data Reduction module led to a notable performance decline (RMSE increased to 9.8; NDCG fell to 0.1394). However, removing the Feature Selection module resulted in a more substantial degradation (RMSE surged to 11.4; NDCG dropped to 0.1312), underscoring that data quality is an even more critical determinant of predictive accuracy. This study ultimately confirms that the synergistic interplay between managing data volume (Data Reduction) and curating data quality (Feature Selection) is indispensable for achieving optimal performance and efficiency.

## 5. Discussion

While this study demonstrates the effectiveness of our DDM framework, it is important to acknowledge the boundaries of our investigation, which in turn delineate promising avenues for future research. The primary limitations are the reliance on a single dataset (FitRec) and the use of only two specific model architectures (DBN and NCF). Therefore, future work should focus on enhancing the framework’s generalizability and expanding its applicability by integrating more advanced strategies.

### 5.1. Latency and Memory Efficiency

Our current approach focuses on an asynchronous retraining cycle triggered by a fixed schedule or the accumulation of new data. We acknowledge the critical need for real-time data processing, particularly in high-stakes healthcare scenarios. Therefore, future research will aim to extend the framework to support real-time scalability. This will involve developing drift-aware conditional updates and integrating incremental and continual learning methods. These enhancements will enable the model to adapt to new data instances as they arrive, thereby minimizing prediction latency and ensuring sustained responsiveness to rapid changes in patient data.

Furthermore, while our study has demonstrated that data reduction and feature selection improve computational efficiency by decreasing the size and dimensionality of the dataset, a more comprehensive future study will investigate memory efficiency from both a data and a model perspective. This research will explore optimizing model architecture to minimize its memory footprint and developing more sophisticated data management techniques that go beyond simple data size reduction. The ultimate goal is to build an end-to-end, memory-efficient pipeline suitable for both resource-constrained edge devices and large-scale cloud environments.

### 5.2. Extending Scalability and Generalizability

The container-based design of our MLOps pipeline renders the proposed methodology inherently scalable. By leveraging Docker and Airflow, the framework facilitates horizontal scaling, ensuring that the retraining pipeline can be seamlessly integrated into large-scale production environments.

A primary limitation regarding generalizability, however, was the dataset itself. While rich in interaction data, the FitRec dataset lacks granular item-side attributes such as target muscle groups, difficulty levels, or potential injury risks. This absence precluded a meaningful evaluation of recommendation diversity and coverage, as the model could not generate recommendations tailored to nuanced fitness goals. Future work should focus on augmenting the dataset with this richer information. Such an enhancement would not only unlock the framework’s potential to generate more diverse and safer workout plans but would also enable a more robust evaluation using these key metrics. To further broaden the framework’s applicability beyond healthcare, subsequent research could also incorporate heterogeneous sensor data to improve its adaptability across other domains.

## 6. Conclusions

This study introduced a Dynamic Data Management (DDM) strategy designed to ensure the long-term performance and efficiency of personalized healthcare recommender systems within an MLOps environment. Our primary contribution lies in the synergistic unification of data reduction and feature selection within a single, automated pipeline. Unlike prior works that address data volume and quality in isolation, our approach simultaneously manages computational load by eliminating redundancy and preserves predictive accuracy by curating the most informative features, enabling proactive adaptation to data and concept drift.

Our empirical evaluation, conducted on the FitRec wearable dataset, validated the effectiveness of this approach. The DDM strategy achieved a 32% reduction in retraining time compared to a full retraining baseline, while maintaining comparable accuracy in both heart rate prediction and recommendation quality. Furthermore, our ablation study confirmed that both modules are critical, and underscored the pivotal role of data quality curated by the feature selection module. These results confirm that DDM is a practical and scalable solution for reducing operational costs without sacrificing performance, offering significant benefits for Edge AI driven healthcare services that demand frequent and efficient model updates.

While the findings are promising, we acknowledge the study’s current scope. Future work will aim to broaden the framework’s generalizability and efficiency. Key directions include (i) extending generalizability by applying the framework to diverse datasets and a wider range of model families and (ii) enhancing efficiency and responsiveness by integrating continual learning to minimize latency and conducting a deeper analysis of memory efficiency for resource-constrained environments. These efforts will advance DDM toward a more flexible and robust solution for the next generation of adaptive recommender systems.

## Figures and Tables

**Table 1:** Packages and versions used in the experiments.

Package	Version of Package
Apache Airflow	2.7.2
Python	3.9
TensorFlow	2.18
Scikit-learn	1.2.2
Docker	27.3.1
Kubernetes	1.32.0

**Table 2:** Results of data reduction.

FitRec Data				
Data Version	Old Data	Reduced Old Data	New Data	Total Data
version 1	-	-	139,161	139,161
version 2	139,161	118,285 (15%)	37,953	156,238
version 3	177,114	144,233 (17%)	37,953	182,186
version 4	215,067	176,339 (18%)	37,953	214,292

**Table 3:** Result of MAE and RMSE.

FitRec Data		
Data Version	MAE	RMSE
version 1	5.2	12.5
version 2	6.7	15.1
version 3	4.5	10.1
version 4	3.9	8.7

**Table 4:** Comparison of recommender model accuracy (K = 10).

Training Phases	Recall	Precision	F1 Score	NDCG
Stage 1	0.25	0.11	0.15	0.0821
Stage 2	0.34	0.14	0.19	0.1134
Stage 3	0.51	0.20	0.28	0.1416
Stage 4	0.52	0.22	0.30	0.1480
Full training	0.54	0.24	0.33	0.1572

**Table 5:** Comparison of Retraining Time by Method (unit: minutes).

FitRec Data			
Data Version	Retraining from Scratch	Simple Data Merging	Ours
version 1	15	15	15
version 2	21	18	17
version 3	25	20	19
version 4	31	24	21

**Table 6:** Ablation study on data reduction (without Feature selection).

Similarity Threshold	Reduction Rate (%)	NDCG	Retraining Time
100%	0%	0.0912	31
99.5%	7%	0.0937	27
99%	14%	0.0924	21
97.5%	30%	0.0908	17
95%	48%	0.0895	14
90%	65%	0.0762	11

**Table 7:** Ablation study on module contribution.

Model	RMSE	NDCG	Retraining Time
DDM	8.7	0.1480	21
Without Data Reduction	9.8	0.1394	24
Without Feature Selection	11.4	0.1312	25

## Data Availability

Restrictions apply to the availability of these data. The data supporting this work were obtained from the FitRec dataset, originally created by Jiamo Ni and collaborators. Access to the dataset can be requested from the authors through https://github.com/nijianmo/fit-rec (accessed on 12 October 2025). The code used for the model implementation and analysis is available from the corresponding author upon reasonable request.
